# Seamless Integration of Conducting Hydrogels in Daily Life: From Preparation to Wearable Application

**DOI:** 10.1002/advs.202306784

**Published:** 2024-01-19

**Authors:** Kusuma Betha Cahaya Imani, Jagan Mohan Dodda, Jinhwan Yoon, Fernando G. Torres, Abu Bin Imran, G. Roshan Deen, Renad Al‐Ansari

**Affiliations:** ^1^ Graduate Department of Chemical Materials Institute for Plastic Information and Energy Materials Sustainable Utilization of Photovoltaic Energy Research Center Pusan National University Busan 46241 Republic of Korea; ^2^ New Technologies – Research Centre (NTC) University of West Bohemia, Univerzitní 8 Pilsen 301 00 Czech Republic; ^3^ Department of Mechanical Engineering Pontificia Universidad Catolica del Peru. Av. Universitaria 1801 Lima 15088 Peru; ^4^ Department of Chemistry Bangladesh University of Engineering and Technology Dhaka 1000 Bangladesh; ^5^ Materials for Medicine Research Group School of Medicine The Royal College of Surgeons in Ireland (RCSI) Medical University of Bahrain Busaiteen 15503 Kingdom of Bahrain

**Keywords:** biomedical applications, Conducting hydrogels, mechanical properties, wearable electronics

## Abstract

Conductive hydrogels (CHs) have received significant attention for use in wearable devices because they retain their softness and flexibility while maintaining high conductivity. CHs are well suited for applications in skin‐contact electronics and biomedical devices owing to their high biocompatibility and conformality. Although highly conductive hydrogels for smart wearable devices are extensively researched, a detailed summary of the outstanding results of CHs is required for a comprehensive understanding. In this review, the recent progress in the preparation and fabrication of CHs is summarized for smart wearable devices. Improvements in the mechanical, electrical, and functional properties of high‐performance wearable devices are also discussed. Furthermore, recent examples of innovative and highly functional devices based on CHs that can be seamlessly integrated into daily lives are reviewed.

## Graphical abstract

Recent significant progress for developing conducting hydrogels is highlighted in this review. Preparation, fabrication, and application of the conducting hydrogels for wearable devices are summarized and discussed for comprehensive understanding. The challenges and opportunities of conducting hydrogels for novel devices are further discussed.

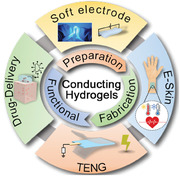

## 1 Introduction

In recent years, significant advances have been made in the field of smart wearable devices, driven by advances in both material systems and device technology. One promising application of wearable devices is to detect fine changes in deformation induced by human motion and convert these changes into electrical signals.^[^ ^1^ ^]^ This capability is beneficial for various applications, such as soft robotics^[^ ^2^ ^]^ and human–machine interfaces.^[^ ^3^ ^]^ Smart wearable devices also offer convenient and noninvasive monitoring of various aspects of human health, such as electrocardiography (ECG) and electromyography (EMG) signals^[^ ^4^ ^]^ as well as drug‐delivery applications in the form of a skin patch.^[^ ^5^ ^]^ As wearable devices become more advanced and sophisticated, the demand for practical and efficient material systems to fabricate high‐performance devices is increasing. To fabricate deformable wearable devices of high performance, highly conductive soft materials are essential to provide an efficient conducting pathway in a conformal shape, which cannot be provided by a metal‐based system.

In particular, CHs have received extensive attention owing to their soft modulus,^[^ ^6^ ^]^ elastic behavior,^[^ ^7^ ^]^ biocompatibility,^[^ ^8^ ^]^ and processability.^[^ ^9^ ^]^ Hydrogels contain a large amount of aqueous medium, which enables biocompatibility, conductivity with electrolytes, and a comfortable interface for monitoring physiological signals. The 3D network structure formed by the chemical cross‐linking of the polymers provides elasticity, which is essential for the recovery of the original shape upon deformation. By manipulating the chemical structure of CHs, their mechanical properties can also be tuned to obtain softness, flexibility, and stretchability, which are important characteristics of conformal and deformable wearable devices.^[^ ^10^ ^]^ Using recently developed advanced techniques, including various 3D printing methods and microfluidic spinning, CHs can be fabricated into controlled shapes and precise geometries.^[^ ^11^ ^]^ Additionally, CHs can be further developed to possess self‐adhesive,^[^ ^12^ ^]^ self‐healing,^[^ ^1^ ^]^ antibacterial,^[^ ^13^ ^]^ anti‐freezing, and anti‐drying^[^ ^14^ ^]^ functionalities, among others. Consequently, CHs have been widely investigated for the development of highly deformable wearable devices.^[^ ^15^ ^]^ Several general, yet in‐depth reviews have summarized the progress of CHs.^[^ ^14^, ^15^, ^16^ ^]^ While these reviews provide valuable insights for understanding specific aspects of CHs, a comprehensive understanding of CHs covering the preparation, applications, mechanism, improvement, and fabrication is demanded for seamless integration of CHs in daily life.

**Figure 1:**
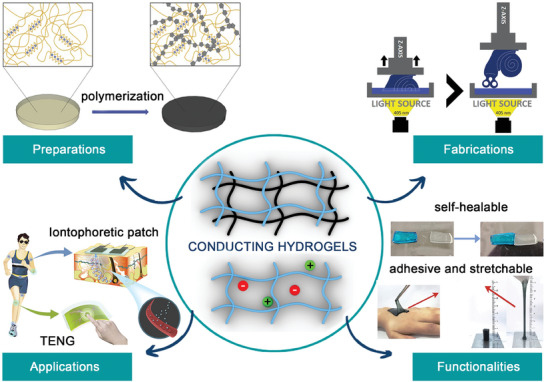
Overview of CH development, including their preparation, fabrication, functionality improvement, and applications. Reproduced with permission.^[^ ^17^ ^]^ Copyright 2022, American ChemicalSociety. Reproduced with permission.^[^ ^4^ ^]^ Copyright 2022, American Chemical Society. Reproduced with permission.^[^ ^119^ ^]^ Copyright 2022, Elsevier. Reproduced with permission.^[^ ^12^ ^]^ Copyright 2020, Springer Link. Reproduced with permission.^[^ ^5b^ ^]^ Copyright 2020, Wiley‐VCH.

In this review, we discuss recent advances in the development of CHs for smart wearable devices. We comprehensively summarize the synthesis of conducting polymers (CPs) and the various approaches used to prepare CHs. We also analyze their properties and discuss the fabrication of specific geometries to improve the performance of the final CH‐based wearable devices, as shown in **Figure** **1**. The studies presented herein contribute to the growing field of wearable devices by highlighting the potential of CHs as versatile and functional material platforms. The development and integration of CHs present challenges and opportunities that highlight the need for novel fabrication techniques and advanced materials.

## 2 Preparation of CHs

CHs are a special class of hydrogels that can conduct electricity owing to the presence of intrinsically conductive materials,^[^ ^6^, ^18^ ^]^ functionalized conductive polymers,^[^ ^19^ ^]^ or conducting ions in insulating polymers.^[^ ^20^ ^]^ CHs comprised of intrinsically electrically conductive materials and cross‐linked polymer networks were categorized in 1995 by Sheppard Jr^[^ ^21^ ^]^ and later by Wallace et al.^[^ ^22^ ^]^ according to their practical applications, including wearable sensors,^[^ ^1^, ^23^ ^]^ biomedicine,^[^ ^24^ ^]^ and actuators.^[^ ^25^ ^]^ Various strategies have been developed to fabricate highly conductive CHs while maintaining their structural integrity. Generally, CHs can be divided into two types: CPs and non‐CPs.

### 2.1 CP‐Based CHs

One approach for preparing CHs is through the use of CPs such as polypyrrole (PPy)^[^ ^1^, ^26^ ^]^ and polyaniline (PANI),^[^ ^7^, ^27^ ^]^ which can be incorporated into the hydrogel network to improve conductivity. In 1999, Osada et al. reported a polythiophene‐based hydrogel synthesized from poly(3‐thiophene acetic acid) (PTAA).^[^ ^28^ ^]^ Later, Bao et al. prepared CH from PANI chains cross‐linked with phytic acid.^[^ ^29^ ^]^ These functionalized polymer hydrogels exhibited good electrical and mechanical properties. Cross‐linked conductive polymers have advantages over traditional conductive materials such as graphite or metal nanoparticles (NPs), as CPs bear various functional groups in their chemical structure.^[^ ^30^ ^]^ For example, the multiple active sites in PANI allow high redox activity and fast kinetic conversion through a dual‐ion mechanism. This property is essential for batteries because it leads to better electrochemical performance at subzero temperatures.^[^ ^30^ ^]^ Two methods are used to prepare CHs from CPs that are generally insoluble in water: in situ polymerization and post‐polymerization.

#### 2.1.1 In Situ Polymerization

**Figure 2:**
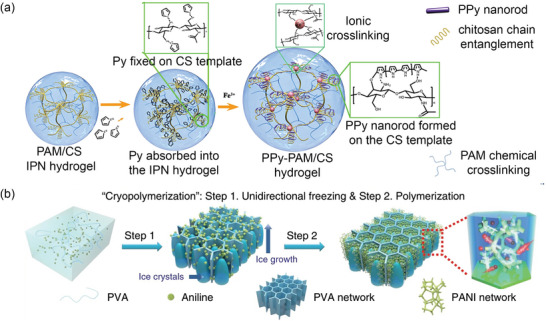
I*n situ* preparation of CHs. a) *Mechanism of in situ* polymerization of PPy on a PAm/CS hydrogel. Reproduced with permission.^[^ ^26^ ^]^ Copyright 2018, American Chemical Society. b) I*n situ* preparation of anisotropic PVA/PANI hydrogels. Reproduced with permission.^[^ ^7^ ^]^ Copyright 2020, Springer Nature.^[^ ^27^ ^]^

In situ polymerization involves the simultaneous cross‐linking of conjugated monomers in a hydrogel matrix. Aniline,^[^ ^7a^ ^]^ pyrrole (Py),^[^ ^26b^ ^]^ thiophene,^[^ ^31^ ^]^ and 3,4‐ethylenedioxythiophene^[^ ^32^ ^]^ were dissolved in a solution containing a cross‐linking agent and redox initiator such as FeCl_3_, ammonium persulfate (APS), and potassium persulfate (KPS). The conjugated monomers underwent oxidative polymerization in the presence of the redox initiator, resulting in the simultaneous formation of a CP network. Lu et al. implemented this method to design CHs with excellent conductivities and mechanical properties.^[^ ^26b^ ^]^ Hydrogels were prepared using chitosan (CS) as the matrix to control the in situ polymerization of the conducting PPy nanorods inside the hydrogel network (**Figure** **2**a). The overall process can be divided into three steps: a) formation of polyacrylamide (PAm)/CS interpenetrating polymer network (IPN) hydrogel by UV photopolymerization, b) introduction of conducting Py monomers into the CS matrix, and c) in situ polymerization of FeCl_3_ on the PAm/CS IPN hydrogel. The resulting hydrogel could withstand a weight of 500 g and was stretched to double its original length.

The preparation of PTAA hydrogels is another example of in situ polymerization.^[^ ^33^ ^]^ The ionizable carboxylic groups on the side chains of PTAA render it water soluble. The resulting hydrogel showed good mechanical strength and an electrical conductivity of 4 × 10^−3^ – 2.0 × 10^−2^ S cm when doped with a 60 wt.% HClO_4_ solution. CHs can be synthesized in matrices such as sodium alginate (SA), poly(vinyl alcohol) (PVA), and agar. In another study, anisotropic PVA/PANI hydrogels with excellent mechanical properties were prepared using modified in situ chemical oxidation polymerization.^[^ ^7a^ ^]^ An aqueous solution of aniline, PVA, and the initiator was frozen unidirectionally to form a 3D‐ordered honeycomb structure, as illustrated in Figure 2b. Aniline was oxidized at low temperatures using APS to form a hydrogel.^[^ ^7a^ ^]^ The synthesis of PANI hydrogels by incorporating phytic acid as a non‐covalent cross‐linker and dopant has also been reported.^[^ ^29^ ^]^ The resulting hydrogel possessed good electrical properties, with a conductivity of 0.23 S cm^−1^.

#### 2.1.2 Post‐Polymerization/Coating

Post‐polymerization/coating involves the immersion of preformed hydrogels in a monomer solution^[^ ^17^, ^34^ ^]^ or an oxidant solution.^[^ ^35^ ^]^ This method can also be used to coat other types of CPs.^[^ ^36^ ^]^ The protonation of functional groups in CPs with acidic dopants such as amino trimethylene phosphonic acid (ATMP),^[^ ^27^ ^]^ H_2_SO_4_,^[^ ^37^ ^]^ and 4‐dodecylbenzenesulfonic acid^[^ ^38^ ^]^ yields high‐performance, pure CHs. For example, ATMP has been used as a cross‐linking agent and dopant to prepare a pure PANI hydrogel^[^ ^27^ ^]^ 2c. ATMP protonates the nitrogen group on PANI by interacting with multiple PANI chains, forming a reticular hydrogel network. ATMP also acts as an excellent acidic dopant owing to its phosphorus group, which increases the hydrophilicity of the CPs. Furthermore, by mixing the oxidation initiator, aniline, and ATMP, a pure hydrogel with a conductivity that was 0.35 S cm^−1^ was obtained. In addition to the use of acidic dopants, another method for obtaining pure CHs involves mixing the volatile additive, dimethyl sulfoxide (DMSO), into a poly(3,4‐ethylenedioxythiophene):polystyrene sulfonate (PEDOT:PSS) dispersion, followed by controlled dry annealing and rehydration. This process resulted in a hydrogel with a conductivity of 40 S cm^−1^, the highest conductivity among all pure hydrogels.^[^ ^19^ ^]^

A stepwise oxidation strategy was employed to prepare pure PPy hydrogels.^[^ ^39^ ^]^ This method involved the addition of Fe(NO_3_)_3_ oxidant to a Py solution and aging it at 25–30 °C for 30 days. The reaction process consists of two steps: a fast and a slow reaction. In the fast‐reaction stage, Fe^3+^ oxidized Py to form PPy. As the Fe^3+^ ions were consumed, the reaction slowed, resulting in the formation of PPy nanowires and nanospheres. The PPy nanostructures then self‐assembled to form a hydrogel network with interconnected pores. The slow‐reaction process allowed the formation of a more stable and uniform hydrogel structure with enhanced electrical conductivity. This stepwise oxidation strategy for the preparation of pure PPy hydrogels has several benefits: a highly porous structure that provides a large surface area for electrode–electrolyte interactions, no need for external dopants, making the hydrogels more biocompatible and environmentally friendly; enhanced electrochemical performance and good mechanical strength, which is crucial for the application of hydrogels in various devices.

Another example of a post‐polymerization method is the synthesis of the PANI‐GelMA hydrogel reported by Soman et al. GelMA hydrogels were first soaked in an oxidizing agent to incorporate APS.^[^ ^40^ ^]^ The APS‐soaked GelMA hydrogels were then incubated in an aniline solution to form the PANI‐GelMA hydrogel. The resulting hydrogel exhibited good electrical conductivity with a resistance of 2.9 kΩ. Another example is the synthesis of electrically conductive and mechanically strong PEDOT. In this case, PSS‐coated GelMA hydrogels were obtained by soaking GelMA hydrogels in a PEDOT:PSS solution.^[^ ^41^ ^]^

### 2.2 Non‐CP‐Based CHs

Non‐CP‐based CHs have gained significant attention in recent years owing to their unique properties, including mechanical flexibility,^[^ ^42^ ^]^ swelling behavior,^[^ ^43^ ^]^ and electrical conductivity arising from the incorporation of conducting fillers.^[^ ^44^ ^]^ Therefore, they are promising candidates for energy storage and conversion applications. These hydrogels can be used as electrodes in supercapacitors and batteries by incorporating conducting fillers and immersing them in electrolytes.^[^ ^10^, ^45^ ^]^ The high surface area of the hydrogel matrix, coupled with its conductivity, allows efficient charge storage and rapid ion transport, leading to enhanced energy‐storage performance. Specific biomolecules or enzymes incorporated into a hydrogel matrix can be used as biosensors for detecting analytes in biological fluids.^[^ ^46^ ^]^ Additionally, these hydrogels can serve as platforms for tissue engineering,^[^ ^47^ ^]^ drug‐delivery systems, and bioelectrodes for neural interfaces, where their electrical conductivity facilitates cell signaling and communication.^[^ ^48^ ^]^ The responsive nature of non‐CP‐based CHs and their conductivity enable their use as actuators in soft robotics.^[^ ^2^, ^49^ ^]^ Applying an electric potential allows the hydrogel to change volume, resulting in mechanical motion.^[^ ^49^ ^]^ This property has been exploited in the development of artificial muscles,^[^ ^50^ ^]^soft grippers,^[^ ^49^ ^]^ and other deformable robotic systems.^[^ ^6^, ^51^ ^]^

In this section, we explore two essential modifications of nonconducting‐polymer‐based CHs: incorporation of conducting fillers and immersion in electrolytes. These strategies are crucial for enhancing the conductivity of these hydrogels and expanding their range of applications.

#### 2.2.1 Incorporation of Conducting Fillers

**Figure 3:**
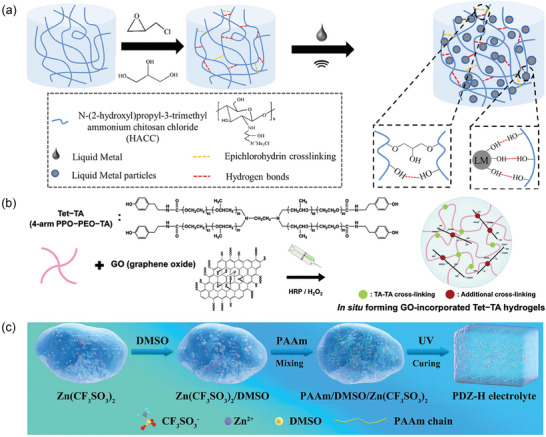
Preparation of non‐CP‐based CHs. a) Illustration of preparation of CHACC‐LM hydrogel through chemical cross‐linking. Reproduced with permission.^[^ ^18^ ^]^ Copyright 2022, Wiley‐VCH. b) Schematic diagram of preparation of Tet‐TA/GO hydrogels through horseradish‐peroxidase‐catalyzed cross‐linking. Reproduced with permission.^[^ ^52^ ^]^ Copyright 2015, Royal Society of Chemistry. c) Preparation of Zn‐dendrite‐free CHs. Reproduced with permission.^[^ ^53^ ^]^ Copyright 2022, Wiley‐VCH.

One of the key strategies for imparting electrical conductivity to non‐CP‐based CHs is the incorporation of conducting fillers. These fillers can be metal‐ or carbon‐based materials, including liquid metals (LMs), silver nanowires (AgNWs), carbon nanotubes (CNT), and graphene. The choice of conducting filler depends on its electrical properties, compatibility with the polymer matrix, and ease of fabrication. The preparation of CHs with a uniform dispersion of metal‐based fillers is challenging because the fillers can solidify within the hydrogels.^[^ ^54^ ^]^ Li et al. uniformly dispersed LM by applying ultrasonic treatment for more than 48 h^[^ ^18^ ^]^ (**Figure** **3**a). Chitosan quaternary ammonium salt (CHACC) was chemically cross‐linked with epichlorohydrin and glycerol in alkaline conditions. The prepared hydrogel was then added to LM and ultrasonicated, resulting in the formation of hydrogen bonds between the CHACC polar groups and the surface oxide of the LM. Finally, the CHACC‐LM hydrogel was formed by heating.

Another example of a metal‐based filler is AgNWs, which can be prepared in a straightfoward manner and exhibit high electrical conductivity, making them suitable conductive fillers. Huang et al. prepared CHs containing AgNWs through a two‐step polymerization method, which increased the cross‐linking density.^[^ ^55^ ^]^ First, a precursor hydrogel was prepared by precipitation polymerization of *N*‐isopropylacrylamide (NIPAm) monomer and *N,N′*‐methylenebisacrylamide (MBAA) cross‐linker, which was initiated by KPS in the presence of sodium dodecyl sulfate (SDS). The addition of SDS prevented the diffusion of monomers and radicals from the precursor hydrogel. The hydrogel then acted as a nano/micro cross‐linker for the second polymerization of NIPAm, producing a highly cross‐linked hydrogel containing AgNWs.

Graphene is one of the most promising conducting fillers. The first graphene‐based hydrogel, reported by Shi et al., was fabricated using a one‐step hydrothermal method.^[^ ^56^ ^]^ In this preparation, graphene oxide (GO) sheets were dispersed in an aqueous solution and then hydrothermally treated. During the hydrothermal process, the GO sheets self‐assembled into a 3D network structure, forming a graphene‐based hydrogel. Park et al. prepared graphene/PPO‐PEO hydrogels with enhanced mechanical properties and electrical conductivity by incorporating graphene or GO into a matrix of PPO and PEO, as illustrated in Figure 3b.^[^ ^52^ ^]^ A tetronic–tyramine (Tet‐TA) solution was mixed with graphene nanomaterials and cross‐linked using a horseradish peroxidase catalyst. The dispersion of GO in the resulting CHs was improved by the molecular oxidation of graphene and the amphiphilic nature of Tet‐TA.

Graphene/hydroxyapatite hydrogels have also been developed for potential applications in tissue engineering by combining graphene with hydroxyapatite, a calcium phosphate compound, to improve their mechanical strength and biocompatibility.^[^ ^57^ ^]^ Graphene/PVA hydrogels were also formed by incorporating graphene into a PVA matrix.^[^ ^58^ ^]^ In another work, CS was combined with graphene to form hybrid hydrogels suitable for various biomedical applications.^[^ ^59^ ^]^

CNTs are another example of a carbon‐based filler for electrical conductivity. The incorporation of CNTs into hydrogel networks is driven by the goal of designing engineered tissue constructs that closely mimic the properties of native electro‐responsive tissues in terms of electrical conductivity^[^ ^50^ ^]^, mechanical integrity^[^ ^60^ ^]^, and nanofibrous architecture^[^ ^61^ ^]^. The high aspect ratio and strong intermolecular interactions of the CNTs enhance the structural stability of the hydrogel, allowing it to withstand mechanical forces more effectively.^[^ ^60^ ^]^ CNT loading promotes the alignment and organization of cells within the hydrogel, leading to improved tissue development and functionality.^[^ ^61^ ^]^ Gelatin hydrogels containing single‐walled CNTs were fabricated and investigated for their potential as CHs for the treatment of myocardial infarction (MI), both in vitro and in vivo.^[^ ^62^ ^]^ The developed CHs provide electrical cues to the damaged myocardium. By mimicking the electrical properties of native heart tissue, these hydrogels can enhance tissue repair and functional recovery after MI. However, the biocompatibility of CNTs in tissue engineering remains a subject of debate, as some studies have shown both positive and negative effects.^[^ ^63^ ^]^

Another dopant which has gained importance in recent years is MXenes, they are two‐dimensional transition‐metal carbides and nitrides with good conductivities and hydrophilicities. The incorporation of MXenes into hydrogels improves their electrical conductivity. One approach involves the direct doping of MXenes into polymer hydrogels. In one example, MXenes were directly doped into a PVA hydrogel to synthesize MXene (Ti_3_C_2_T*_x_*)/PVA hydrogel with improved conductivity, self‐healing ability, and mechanical properties compared with the unmodified PVA hydrogel.^[^ ^64^ ^]^ Another approach is to use inorganic materials, such as GO, as gelling agents to improve the interactions with the surface of the MXene and reduce self‐stacking. A graphene‐wrapped MXene obtained by plasma exfoliation demonstrated double the specific capacitance and excellent charge/discharge properties and mechanical stability compared with the bare MXene.^[^ ^65^ ^]^

#### 2.2.2 Immersion in Electrolytes

Enhancing the conductivity of non‐CP‐based CHs by immersing them in an electrolyte solution is a promising approach that has garnered significant attention in recent years. This method leverages the presence of mobile ions in the electrolyte to facilitate the transport of electrical charge within the hydrogel network.^[^ ^43^, ^53^, ^66^ ^]^ In this approach, electrolytes are prepared by dissolving polymers and salts in aqueous media. with LiCl)has been widely used in the preparation of ionic hydrogels because it is more conductive than other salts such as NaCl and KCl owing to the facile migration of Li^+^ ions.

Ionic CHs are typically transparent, and the absence of nanoparticulate phases makes them advantageous for biomedical applications.^[^ ^67^ ^]^ Zhang et al. prepared CHs with a high transparency of >90% at 550 nm using a simple method. First, carboxymethyl chitosan (CMC) was dissolved in water, and epichlorohydrin was added as a chemical cross‐linker. After the hydrogels were formed, they were immersed, and Ca^2+^ ions were introduced to improve their conductivity.^[^ ^68^ ^]^ In another work, Xu et al. prepared Zn‐dendrite‐free hydrogels for battery applications^[^ ^53^ ^]^ (Figure 3c). As a Zn‐ion source, Zn(CF_3_SO_3_)_2_ was mixed with acrylamide (Am) in water and DMSO. Later, APS and MBAA were added to the solution, and the resulting solution was poured into a mold, which was then exposed to UV light to initiate photopolymerization. The negative charge on the PAm chains can facilitate coordination with Zn ions, which restricts the cusp effect and the accumulation of Zn. Consequently, Zn‐dendrite growth can be suppressed during repeated charge/discharge processes. In addition, DMSO suppressed Zn‐dendrite formation by reducing DMSO solvation.

Polyelectrolytes are also used to prepare ionic hydrogels. In general, polyelectrolyte complexes are commonly polymerized and assembled from ionizable monomers^[^ ^69^ ^]^ and natural polysaccharides.^[^ ^70^ ^]^ Following a similar approach, polyelectrolyte hydrogels are formed by cross‐linking and fixing the networks of polyelectrolytes, which simulates an ion‐selective permeation membrane in the presence of free‐moving ions. This system mimics ionic conduction caused by selective and directional salt‐ion diffusion owing to the presence of a concentration gradient between two or more chambers. Poly(acrylic acid) (PAAc), which possesses –COOH groups that can easily ionize H^+^ in the presence of water, is a common polyelectrolyte used in the formation of ionic hydrogels. Therefore, H^+^ can move freely in the 3D hydrogel structure because the negatively charged PAAc chains are fixed. This hydrogel exhibited directional H^+^ diffusion under compression, proving its use as a mechanical–electrical converter.

Polyzwitterionic hydrogels have been employed to enhance the electrochemical performance of electrolyte‐based CHs. Xie et al. reported the preparation of a zwitterionic hydrogel through free‐radical polymerization of the zwitterionic monomer propylsulfonate dimethylammonium propylmethacrylamide (PPDP).^[^ ^43^ ^]^ After the polyzwitterionic hydrogel was formed, it was immersed in an LiCl solution to acquire conductivity. PPDP allowed for high water retention surrounding the charged groups and provided ion‐migration channels for electrolyte ions, improving the electrochemical performance of solid‐state supercapacitors.

Different electrolytes possess varying ionic mobilities and interact differently with hydrogels, thereby influencing their overall conductivity.^[^ ^42^, ^53^, ^68^ ^]^ Additionally, the concentration of the electrolyte solution must be carefully controlled to achieve the desired conductivity without compromising the mechanical and structural integrity of the hydrogel.^[^ ^71^ ^]^

Despite their various advantages, non‐CP‐based CHs still face some challenges, such as the need to improve their long‐term stability and durability. The degradation of conducting fillers,^[^ ^72^ ^]^ ion leaching,^[^ ^73^ ^]^ and mechanical fatigue^[^ ^74^ ^]^ can affect the performance and reliability of these materials. Overcoming the challenges related to conductivity optimization, long‐term stability, biocompatibility, and scalability will pave the way for their successful integration into practical devices and systems.

### 2.3 Gradient CHs

Gradient structures, characterized by a gradual change in properties or structure along a specified direction, have garnered significant attention in materials science. Various researchers have developed innovative materials that employ gradient structures to enhance specific functionalities. For instance, Wu et al. developed a conductive hydrogel with a gradient structure, employing a soaking strategy.^[^ ^75^ ^]^ They induced a concentration gradient, enabling conductive monomers to permeate from the surface to the interior of the hydrogel. The depth of the conductive networks was adjusted by manipulating the soaking time. This manipulation resulted in a gradient structure, where the conductive layer decreases toward the nucleus of the conductive hydrogel. The hydrogels were synthesized by blending a CS and isocyanate‐terminated‐methoxy‐polyethylene glycols solution with Am monomer and MBAA as a cross‐linker. Subsequently, the hydrogel obtained was immersed in an acidic Py solution to achieve a surface coated with PPy. This material demonstrated remarkable attributes, including ultrahigh stretchability (4000%), exceptional transparency (90%), and a skin‐like Young's modulus of 40 kPa.

Wu et al. also used a similar method to synthesize a conductive hydrogel.^[^ ^76^ ^]^ The hydrogels were synthesized using a free radical solution polymerization method, with Am as the monomer, and waterborne hexamethylene diisocyanate trimer and MBAA as cross‐linkers. To achieve the gradient structure, the hydrogels underwent sequential immersion in acidic Py and APS solutions, which resulted in the formation of a PPy coating on the surface of the hydrogel. The resulting CH exhibited outstanding characteristics, with a strain exceeding 900% and a Young's modulus of 8 kPa.

In another work, Wu et al. prepared a CH by incorporating conductive montmorillonite (MMT) NPs.^[^ ^77^ ^]^ MMT were first immersed in a Py solution. Then the absorbed Py was in situ polymerized. The resulting conductive montmorillonite nanoparticles (CMMT) were added to a suspension of 2‐hydroxyethyl methacrylate and Am. To achieve different levels of cross‐linking, a solution of MBAA was then added. The gradient effect was achieved through meticulous adjustment of the CMMT concentration, gradually transitioning from the top surface to the bottom of the material. This gradient design led to a progressive change in properties, transitioning, for instance, from conductive to non‐conductive characteristics. The resulting hydrogel showcased remarkable mechanical properties, with an impressive strain capability of 9000% and a Young's modulus of 60 kPa.

## 3 Fabrication of CHs

The fabrication of CHs with controlled shapes and dimensions is necessary for practical applications. In addition to its physicochemical properties, a material's shape and structure is closely related to its function.^[^ ^6^, ^78^ ^]^ By mimicking the various shapes of human body parts or natural organisms, hydrogels can efficiently realize specific functions for applications in soft robotics, electronics, and biomedical engineering. CHs are traditionally prepared using a mold, making it difficult to obtain functional shapes and micro‐ or nanostructures. To overcome this problem, numerous research groups have reported efficient methods, including direct ink writing (DIW),^[^ ^11^, ^79^ ^]^ stereolithography (SLA),^[^ ^80^ ^]^ digital light processing (DLP),^[^ ^4^, ^81^ ^]^ inkjet printing,^[^ ^29^, ^82^ ^]^ microfluidic spinning,^[^ ^66^, ^83^ ^]^ and electrospinning^[^ ^44^, ^84^ ^]^ to fabricate CHs at high resolutions. In this section, we summarize the cutting‐edge methods used to fabricate CHs with the desired shapes.

### 3.1 DIW

DIW is a printing method in which a computer controls the movement of a platform or printhead, and ink with a specific viscosity range is extruded at the desired position to prepare a 3D structure. Hydrogels are typically printed as precursors and then polymerized solidify their shape.^[^ ^85^ ^]^ DIW generally works with pressure‐driven extrusion techniques for precured hydrogels, in which a syringe typically attached to a pump allows for the controlled deposition of 3D layers through a nozzle according to the target shape.^[^ ^11a^ ^]^ A notable feature of DIW is the range of viscosities suitable for 3D printing. The viscosity should be sufficiently low for extrusion but not so high that it clogs the nozzle.^[^ ^11a^ ^]^ In addition, the ink should have a shear‐thinning property, that is, upon shearing force, the ink assumes a liquid state, whereas after printing, it should immediately return to the solid state, where it can maintain the desired structure.^[^ ^79a,79d^ ^]^ Despite these requirements, given the low cost of producing devices and the relatively simple underlying concept, DIW has been extensively used to produce CHs. In addition, DIW allows the printing of many materials with various physicochemical properties using a single printer by adding cartridges.

**Figure 4:**
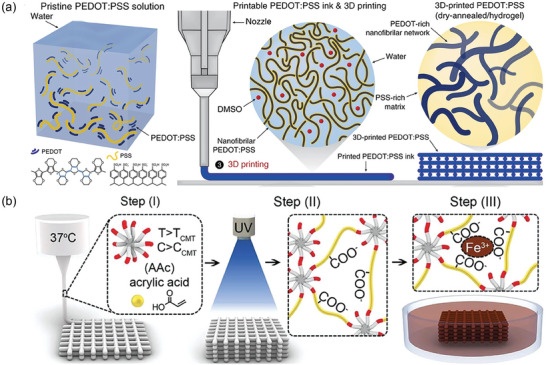
a) Schematic illustration of DIW 3D printing of PEDOT:PSS through lyophilization and redispersion process. Reproduced with permission.^[^ ^79a^ ^]^ Copyright 2020, Springer Nature. b) DIW 3D printing of Pluronic F127‐dimethacrylate via micelle formation and additional ionic bond formation between polyacrylic acid (PAAc) and iron ions. Reproduced with permission.^[^ ^11a^ ^]^ Copyright 2021, Wiley‐VCH.

As discussed in Section 2, PEDOT:PSS is a commonly used polymer mixture for preparing CHs. Zhao et al. treated an aqueous PEDOT:PSS solution by cryogenic freezing, followed by lyophilizing and redispersing it in water and DMSO^[^ ^79a^ ^]^ (**Figure** **4**a). As a result, the CPs possessed a high resolution and a high aspect ratio along with remarkable 3D printability.

Yoon et al. used Pluronic F127‐dimethacrylate, a triblock copolymer that can form micelles above its critical micelle temperature and concentration and possesses a shear‐thinning property suitable for 3D printing, as a platform to print various hydrogels (Figure 4b). PEDOT:PSS was incorporated into the pre‐gel solution with AAc to introduce conductivity.^[^ ^11a^ ^]^ The 3D‐printed structure was then fixed by photopolymerization and then immersed in an iron solution to enhance its mechanical properties by ionic cross‐linking with AAc, which yielded biocompatible, tough, and conductive 3D printed hydrogels.

In another study, Boccaccini et al. prepared electrically conductive, cytocompatible, and 3D printable hydrogels scaffolds by combining Py and oxidized alginate–gelatin (ADA‐GEL) hydrogels.^[^ ^79b^ ^]^ Inside the ADA‐GEL matrix, electroactive PPy:PSS was prepared by oxidizing Py followed by dual cross‐linking with Ca^2+^. The formation of PPy inside the hydrogels increased the stiffness and electrical conductivity, which affected the cell–material interaction. In addition, porosity was introduced using 3D printing, and the seeding efficiency was increased. The combination of 3D printability and enhanced conductivity enables its application in cartilage tissue engineering.

CHs can also be used to fabricate advanced wearable sensors via 3D printing. For example, Vlassak et al. used hydrogels embedded in a polydimethylsiloxane (PDMS) elastomer to fabricate a resistive‐type strain gauge.^[^ ^79d^ ^]^ For the 3D‐printable hydrogel precursor, two components—a rheological modifier and a hydrogel component—were mixed. To prepare the rheological modifier, an uncross‐linked PAm solution was prepared using photopolymerization, while the hydrogel component was prepared by combining an Am pre‐gel solution LiCl, an ionic hygroscopic salt. The rheological modifier and hydrogel components were mixed using centrifugation. 3D printing facilitated the integration of hydrogels and elastomers to prepare a resistance strain gauge in the shape of a single loop.

### 3.2 SLA

SLA is a 3D printing method in which a laser beam is used to solidify a pre‐gel solution. It controls light movement to form a pattern on the precursor solution; the solution platform is gradually lowered to irradiate the solution layer by layer, finally preparing a 3D structure.^[^ ^86^ ^]^ The resolution depends on light exposure, which is the energy passed through the solidifying area. During printing, the light intensity decreases as the penetration depth increased, which may result in incomplete polymerization.^[^ ^87^ ^]^ Therefore, the method is limited by the cure depth, which is the polymerization limit at which the precursor solution remains in the liquid state.^[^ ^87^ ^]^ Despite this limitation, SLA is suitable for preparing CHs because it provides high‐resolution printing with minimal processing difficulties.^[^ ^80d^ ^]^

**Figure 5:**
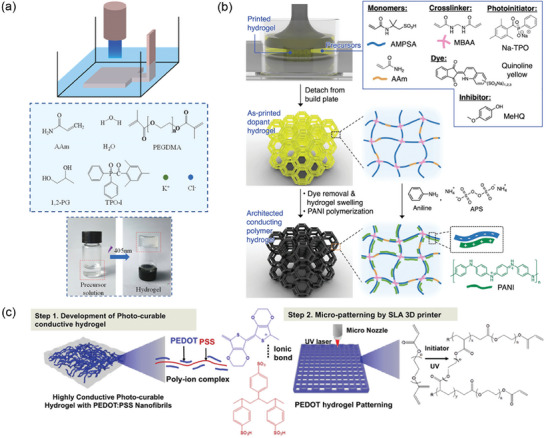
Schematic illustration of the SLA 3D printing of a) Am‐based CHs in potassium chloride (KCl) along with the chemical structure of the precursor solution. Reproduced with permission.^[^ ^80a^ ^]^ Copyright 2022, Elsevier. b) 3D PANI hydrogels produced from aqueous precursor solution and their chemical structure. Reproduced with permission.^[^ ^80b^ ^]^ Copyright 2021, Royal Society of Chemistry. c) Micropattern of photocurable PEDOT:PSS with polyethylene glycol diacrylate (PEGDA). Reproduced with permission.^[^ ^80c^ ^]^ Copyright 2019, Elsevier.

Wang et al. prepared degradable hydrogels using a projection micro‐SLA to develop a wearable motion sensor and transient circuit^[^ ^80a^ ^]^ (**Figure** **5**a). The hydrogels consisted of Am, poly(ethylene glycol) dimethacrylate, and KCl. The conductivity was determined in the presence of KCl. When placed in an alkaline environment containing hydroxide ions, the aliphatic groups of the gel degraded, disintegrating the 3D network. Furthermore, PAm was hydrolyzed under alkaline conditions, converting the amide group into a carboxyl group, and then neutralized into sodium carboxylate by Na^+^ and OH^–^ ions. Owing to its conductivity and degradability, the hydrogel could be used to fabricate a programable human–machine interface by capturing an electromyography signal from a human arm.

Owing to the architecture of SLA, its electrical properties can also be tuned, as reported by Wang et al.^[^ ^80b^ ^]^ The SLA precursor contains an anionic monomer for conductive network template growth and monomers with a remarkable swelling ratio, leading to the formation of a sufficiently strong hydrogel under compression. Therefore, PANI was chosen as the CP, and 2‐acrylamido‐2‐methyl‐1‐propanesulfonic acid and Am were selected as monomers. From these materials, 3D‐printed hydrogel lattices were prepared, resulting in a stress–strain plateau that overlapped with the strain, which enhanced the stability (Figure 5b).

In another study, Zhang et al. exploited the SLA architecture to improve neuronal differentiation through the systematic transfer of electrical stimulation to encapsulated cells.^[^ ^80c^ ^]^ To prepare photocurable hydrogels for SLA printing, PEDOT:PSS was frozen, lyophilized, and dissolved in water and ethylene glycol (EG). Subsequently, poly(ethylene glycol) diacrylate (PEGDA) and a photoinitiator were added to the mixture (Figure 5c). The mechanical properties of the hydrogel improved significantly owing to the stiff PEGDA polymer network, whereas PEDOT:PSS slightly decreased the stress owing to hindered UV‐light penetration during curing. However, PEDOT:PSS increased cell adhesion and the number of living cells. In addition, 3D printing enables the production of a well‐integrated scaffold with a predesigned geometry.

Giannelis et al. prepared ionically conductive and 3D‐printable hydrogels.^[^ ^80d^ ^]^ To enable fast 3D printing at high resolution, SLA resins must be polymerized rapidly using a precursor solution with low viscosity. In this study, Am was mixed with the photoinitiators riboflavin and triethanolamine; [2‐(acryloyloxy)ethyl]trimethylammonium chloride (AETA), which is a cross‐linker; and ionic sulfonate‐modified silica NPs. The sulfonate groups react with the quaternary ammonium groups in the polymer network. Although Am exhibited slow polymerization kinetics, the reaction speed was promoted in the presence of AETA. The addition of silica NPs also improved the gelation speed owing to the physical entanglements generated in the hydrogel composite. In addition, a high ionic conductivity was achieved in the presence of AETA and silica NPs, which added counterions to the solution.

### 3.3 DLP

DLP is another 3D‐printing method based on photopolymerization that provides high resolution.^[^ ^81a^ ^]^ However, unlike in SLA, where a laser is used to polymerize the liquid resin point by point, in DLP, an entire layer is polymerized using a digital projector at the bottom combined with a moving platform on top, resulting in fast printing.^[^ ^4^, ^26^ ^]^ Since the printed part is not immersed in the resin solution, swelling might not be a problem in SLA printing. In addition, multimaterial printing with DLP is possible because the ink can be easily exchanged.^[^ ^81c^ ^]^ Since fast gelation is required for DLP, material selection is limited. The printed part may also be potentially damaged owing to the disturbance of the polymerization upon oxygen exposure.

**Figure 6:**
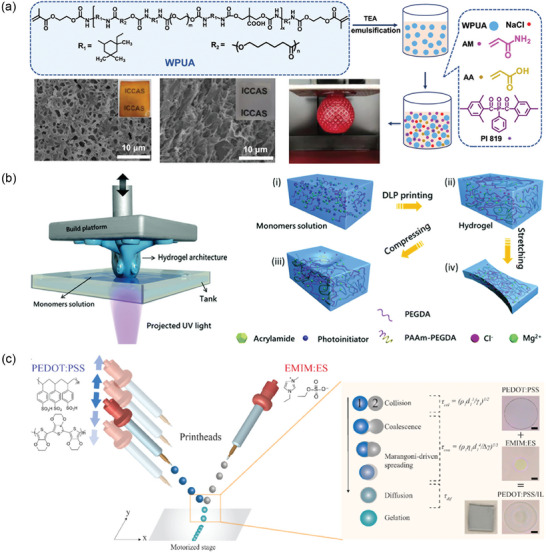
Schematic illustration of DLP printing. a) Microemulsion processing of P(UA‐*co*‐Am‐*co*‐AAc) hydrogel and SEM images for resulting (left) cured hydrogel and (right) hydrogel post‐processed with ferric ions. Reproduced with permission.^[^ ^81a^ ^]^ Copyright 2021, American Chemical Society. b) Formation mechanism of PAm‐PEGDA hydrogels. Reproduced with permission.^[^ ^81b^ ^]^ Copyright 2019, Royal Society of Chemistry. c) Scheme showing the mechanism of microreactive inkjet printing with PEDOT:PSS and EMIM:ES. Reproduced with permission.^[^ ^82^ ^]^ Copyright 2019, American Chemical Society.

Huang et al. prepared DLP‐printed CHs to fabricate microstructured pressure sensors.^[^ ^81a^ ^]^ Their study addressed the photoinitiator problem, that is, the tendency for hydrophobic photoinitiators to have poor solubility and a hydrophilic photoinitiator to have low efficiency. This problem was solved by synthesizing a microemulsion of waterborne polyurethane acrylate to incorporate the hydrophobic photoinitiator (**Figure** **6**a). Subsequently, Am and AAc were added to obtain a photosensitive resin, and metal‐ion coordination enhanced the mechanical properties. The conductivity of the hydrogel originated from the carboylate and ferric ions in the network. DLP printing can achieve a microstructured pressure sensor with twice the sensitivity of a planar sensor.

Wang et al. fabricated a sensor by sandwiching a VHB 4905 adhesive tape between the hydrogels and connecting it to the electrodes.^[^ ^81b^ ^]^ The hydrogels were prepared by copolymerizing Am and PEGDA. Although PAm is a flexible hydrogel with a high water‐intake capacity, PEGDA improves the toughness of the hydrogel (Figure 6b). Endowed with both characteristics, the resulting hydrogels showed enhanced toughness and elasticity and were capable of returning to their original shapes upon deformation. Magnesium chloride was introduced to impart ionic conductivity and water retentiveness to the hydrogel.

The DLP‐printed CHs were ECG and EMG devices, as demonstrated by Mecerreyes et al.^[^ ^4^ ^]^ The inks were prepared by dispersing PEDOT:PSS in a photocurable matrix containing PEGDA and vinyl monomers. To improve conductivity, EG was used as a dopant for PEDOT:PSS. EG screens the ionic interactions between PEDOT and PSS through hydrogen‐bond formation with PSS. As a result, PEDOT and PSS separated, enabling a PEDOT chain in a linear orientation. The DLP‐printed sample was then used as the working and counter electrodes for ECG and as the working electrode for EMG. The results show that both the ECG and EMG recordings were superior to those generated using standard medical electrodes.

### 3.4 Inkjet Printing

Inkjet printing is a type of 3D printing in which materials are deposited as droplets to achieve the desired structure. The printer typically comprises a platform, curing devices, and jetting heads. The inks are ejected through piezoelectric, thermal, or electrostatic processes.^[^ ^88^ ^]^ During the printing process, the heads move in 2D directions, whereas the stage moves along the Z‐axis to stack the construction.^[^ ^89^ ^]^ In general, there are two types of inkjet printing: continuous inkjet printing and drop‐on‐demand printing.^[^ ^90^ ^]^ In continuous inkjet printing, the inks are continuously pushed to form droplets through Rayleigh instability, whereas droplets are formed by a piezoelectric or thermal trigger in drop‐on demand. For inkjet printing, the ink requires a low viscosity and shear thinning, which means that the gelation speed is crucial for preventing spreading after the ink is deposited.^[^ ^82^ ^]^ Therefore, surface tension is also essential for the formation of droplets because excessively high surface tension leads to the breakage of the droplets during the printing process.^[^ ^91^ ^]^ Inkjet printing has the advantage of high resolution and provides multimaterial printing by adding printheads.^[^ ^92^ ^]^ These advantages make inkjet printing ideal for 3D printing conductive materials.

Stringer et al. used microreactive inkjet printing (MRIJP) to print PEDOT:PSS.^[^ ^82^ ^]^ The MRIJP works using two printheads, where each printhead deposits a monomer and an agent that enables the incorporation of both materials into the substrate and form the desired structure (Figure 6c). PEDOT:PSS was used as the monomer, and the ionic liquid 1‐ethyl‐3‐methylimidazolium ethyl sulfate (EMIM:ES) was used as the polymerizing agent. Collisions between PEDOT:PSS and EMIM:ES allow the formation of hydrogels and removes the Marangoni flow between the substrate and reactants. Using this method, the large‐scale patterning of hydrogels and both 2D and 3D structures can be achieved. In addition, the resulting PEDOT:PSS conductivity was indistinguishable from that of the sample prepared by spin coating.

In another study, Bao et al. prepared CHs by reacting PANI with phytic acid.^[^ ^29^ ^]^ For the inkjet printing process, a solution containing an oxidative initiator was printed, and then a solution containing aniline monomer and phytic acid was printed. Gelation occurred by the protonation of PANI nitrogen groups and the subsequent reaction of phytic acid with several PANI chains, creating a mesh‐shaped hydrogel network. Inkjet printing was performed without additives, resulting in hydrogels with a low impedance and high capacity for energy storage.

### 3.5 Microfluidic Spinning

Microfluidic spinning allows the manipulation of liquids through microscale channels.^[^ ^93^ ^]^ Recently, microfluidic spinning was used to prepare CHs because it is a simple method to prepare microsized hydrogels. Using microfluidic technology, hydrogels with various shapes, such as microfibers^[^ ^66^, ^83^ ^]^ and hollow microtubes,^[^ ^11^, ^94^ ^]^ can be prepared, as well as spheres,^[^ ^95^ ^]^ Janus,^[^ ^96^ ^]^ and core–shell^[^ ^97^ ^]^ structures. For fibrous hydrogels, microfluidic spinning is superior to other traditional methods, such as tube molding, owing to the possibility of large‐scale production with a controlled uniform diameter in a short time.^[^ ^11^, ^94^ ^]^ In addition, the dimensions of the fibers can be easily tuned by adjusting both the diameter of the microchannels and the flow rate of the pre‐gel solution.^[^ ^66^, ^83^ ^]^ The microfluidic system usually involves the polymerization of two or more precursor solutions flowing in a channel, which requires a fast solidification process at the junction point. For this system, various geometries can be prepared, and different materials can be incorporated, allowing for the facile preparation of CHs.

**Figure 7:**
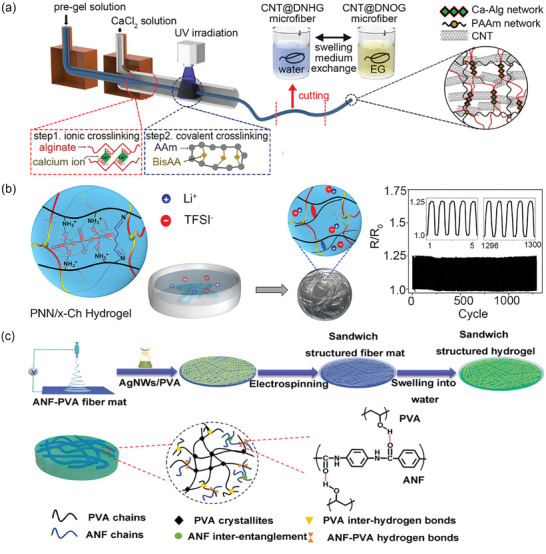
Schematic illustration of a) microfluidic preparation of hydrogel microfiber containing CNTs and resulting double‐network structure. Reproduced with permission.^[^ ^83b^ ^]^ Copyright 2020, American Chemical Society. b) (left) Solvent exchange of poly(*N*‐isopropylacrylamide‐*co*‐*N,N’*‐diethylacrylamide/chitosan) (poly(NIPAm‐*co*‐NDEAm/CS) hydrogel into ionic microfiber, and (right) photographs of LED power source connected with microfiber stretched to various lengths. Reproduced with permission.^[^ ^66^ ^]^ Copyright 2022, Wiley‐VCH. c) Illustration of electrospinning of the sandwich structure comprising layers of PVA–aramid nanofibers (ANF) and a layer of silver nanowires (AgNWs)/PVA. Reproduced with permission.^[^ ^44^ ^]^ Copyright 2021, Wiley‐VCH.

Yoon et al. prepared hydrogel microfibers containing conducting materials such as PEDOT:PSS^[^ ^83a^ ^]^ or CNTs^[^ ^83b^ ^]^ using a microfluidic system. To prepare the microgel, a pre‐gel solution containing Am, alginate, and a conducting filler was injected coaxially with another solution containing CaCl_2_ (**Figure** **7**a). When the solutions meet, a microfiber is formed through ionic bonding between calcium and alginate, and the monomers are then photopolymerized to form double‐network hydrogels containing conducting materials. Depending on the application, the solvent can be exchanged with EG to prevent drying of the medium. During the microfiber preparation process, the CNTs flowed in parallel owing to the shear forces in the microfluidic capillary, which led to the formation of an effective percolation network. As a result, the microgel showed negligible hysteresis after 1000 cycles of stretching owing to the CNT alignment along the fiber direction.

In another study, Yoon et al. developed CHs using a coaxial flow of pre‐gel solution and cold water for photopolymerization in a fiber shape based on the solubility difference.^[^ ^66^ ^]^ The monomers were composed of NIPAm, *N,N*’‐diethylacrylamide (NDEAm), a cross‐linker, a photoinitiator, and CS, and acetic acid was added to dissolve CS. Monomer mixtures were injected into the inner capillaries, and cold water was injected into the outer capillaries. When cold water contacted the monomer solution at the junction, CS readily solidified owing to the solubility difference, which depended on the temperature and coagulation resulting from chain entanglement.

CS acted as a template, while NIPAm and NDEAm were simultaneously photopolymerized to form microfiber hydrogels. The microfibers were reinforced in tripolyphosphate (TPP) and GA solutions through a Schiff base reaction between the aldehyde groups in GA and amine groups in CS, forming imine bonds. Ionic cross‐linking then occurred between the protonated amines from CS and the multivalent anions from TPP. To impart conductivity to the microfiber, the microfiber was immersed in a lithium bis(trifluoromethanesulfonyl)imide solution. The resulting microfiber demonstrated superior conductivity, as it was kept continuously wet and exhibited constant resistivity under elongation (Figure 7b).

Zhao et al. used coaxial flow to prepare microfiber hydrogels by injecting PEDOT:PSS and H_2_SO_4_ into an inner Janus capillary.^[^ ^83c^ ^]^ SA was injected into the outer capillary for the fast gelation of alginate. Simultaneously, the PEDOT chain coil expanded in the acidic medium, partly removing PSS from the complex. The acidity ratio determined the conductivity of the resulting microfiber hydrogel. This led to the formation of physical cross‐links between the PEDOT chains, and CaCl_2_ collected microfibers containing PEDOT, thereby reinforcing the gelation of alginate. The alginate shell provides time for the reaction between PEDOT:PSS and H_2_SO_4_ to occur.

Recently, Zhao et al. developed hydrogels comprised of GO and hybrid dextran (DEX) using histidine.^[^ ^83^ ^]^ The Knoevagel condensation between the cyanoacetate and benzaldehyde groups in the DEX polymer was hindered by the presence of GO owing to the acidic conditions. However, GO acted as a promoter because the pH normalized with the addition of histidine. GO and histidine form a complex in aqueous media, accelerating the formation of carbon double bonds via the Knoevagel condensation reaction. This property was exploited to prepare microfibers using a microfluidic system, in which DEX and GO were injected into a histidine solution. In the presence of GO above the concentration where rapid gelation occurs and below the concentration where the nozzle is clogged, uniform fibers could be prepared. The microfiber hydrogels exhibited excellent conductivity owing to the presence of GO, which can be used as a flexible motion sensor.

### 3.6 Electrospinning

Electrospinning is a technique used to produce polymeric fibers from a liquid using an electrostatic force. This is also an effective method for preparing fiber‐shaped CHs, but their diameter is typically on the nanometer scale.^[^ ^98^ ^]^ Electrospinning typically involves a power supply of direct or alternating current, a needle, a syringe pump, and a collector.^[^ ^84a^ ^]^ Electrospinning is initiated by applying an electrical voltage that extends the polymer solution from the droplets to the fibers. Droplets are formed by surface tension, and electrification deforms them into Taylor cones.^[^ ^99^ ^]^ A charged jet is ejected from the Taylor cone, and the jet movement changes from a straight line to a whipping motion caused by bending instabilities. This process continues until a fiber shape is formed. Polymerization generally occurs during fiber formation when the precursor is exposed to light or heat after injection. The prepared fibers are then deposited in a collector. Various materials in the form of solutions, melts, or small molecules can be processed using electrospinning.^[^ ^100^ ^]^ This simple setup makes electrospinning an attractive method for preparing CH fibers.

Zhu et al. prepared hybrid hydrogels with sandwich structures comprising layers of aramid nanofiber (ANF)‐reinforced PVA (ANF‐PVA) andAgNW‐loaded PVA (AgNW/PVA).^[^ ^44^ ^]^ (Figure 7c) Electrospinning was performed to prepare the ANF‐PVA fiber mats. Then, sandwich‐structured CHs were prepared by vacuum filtering AgNW/PVA on top of the fiber mats, followed by electrospinning the ANF‐PVA fiber mat layer on top of it. Owing to the high hydrophilicity of PVA, hybrid hydrogels can be obtained by swelling fiber mats in water. The resulting ANF‐PVA hybrid hydrogels exhibited high toughness, high modulus, and high tensile strength owing to the robust hydrogen bonds between the ANFs and PVA molecular chains, the aligned nanofiber structures, and the enhanced crystallinity of the structures.

Similarly, Oréfice et al. prepared a fast‐response soft actuator using electrospun hybrid hydrogels.^[^ ^84^ ^]^ AAc, Am, and PEGDA were used as the hydrogel precursors, whereas PANI was selected as the conjugated polymer precursor. PVA was also used to increase the viscosity of the solution during the electrospinning process. Electrospun hybrid hydrogels were prepared via the simultaneous photopolymerization of the pre‐gel solution and aniline. Fast electroactive actuation was achieved owing to the high conductivity of PANI, the short diffusion path of the nanofibers, and the swelling of the hydrogel fibers.

In another study, Heuzey et al. prepared hybrid hydrogels by polymerizing PANI in poly(*N*‐isopropylacrylamide) (PNIPAm) hydrogel fibers.^[^ ^84b^ ^]^ A PNIPAm precursor in dimethylformamide was electrospun to form a fiber mat, which was then photopolymerized. The swollen PNIPAm hydrogel fibers were moved to 40°C water to shrink the hydrogels, then transferred to a solution containing phytic acid, aniline, and water at 4°C. Finally, the solution was transferred to an APS solution to polymerize aniline and introduce conductivity. The fibrous structure helped facilitate mass exchange, which preserved PANI in the hydrogel domain, imparting conductivity without sacrificing the thermo‐responsiveness of PNIPAm.

## 4 Improvement of CH Properties

**Figure 8:**
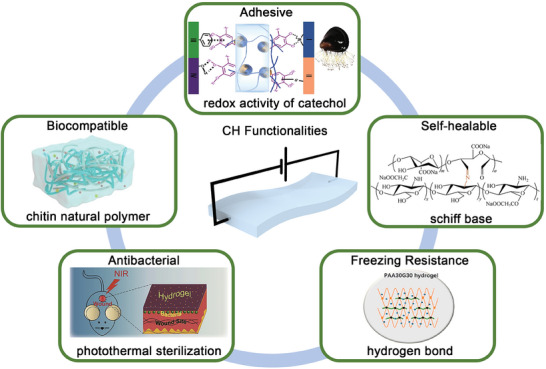
Approaches used to improve hydrogel functionalities. Reproduced with permission.^[^ ^12a^ ^]^ Copyright 2020, Springer Link. Reproduced with permission.^[^ ^104^ ^]^ Copyright 2023, Elsevier. Reproduced with permission.^[^ ^177^ ^]^ Copyright 2020, Elsevier. Reproduced with permission.^[^ ^9^ ^]^ Copyright 2020, Elsevier. Reproduced with permission.^[^ ^111^ ^]^ Copyright 2022, Springer Link.

CHs have a broad range of applications ranging from electrical to biomedical fields.^[^ ^101^ ^]^ Each final application requires the CH to possess specific properties. For example, hydrogels used in wearable sensors^[^ ^102^ ^]^ should have high mechanical flexibility, satisfactory self‐healing performance, and good electrical conductivity, whereas hydrogels used in human‐tissue‐repair systems^[^ ^103^ ^]^ require excellent biocompatibility. Several important requirements of CHs for real‐world applications are illustrated in **Figure** **8** and summarized in **Table** **1**. CHs must be adhesive, cabaple of self‐healaling, resistant to freezing, and possess antibacterial and biocompatible properties.

**Table 1:** Summary of strategies followed to improve various hydrogel properties.

Improved properties	Strategy	Type of hydrogels	Constituents	Improved parameters	References
*Mechanical*	NPs as cross‐linking agents	Polymer NP‐cross‐linked hydrogel	PANI‐*g*‐MeGC Nps PAm	Elongation at break = 400%	[105]
		NP‐cross‐linked hydrogel	DACS Nps Collagen	Tensile modulus = 6.5 kPa Structural stability = compression up to 200 g	[105]
		Non‐covalent micelle cross‐linked hydrogel	PEG, UPy	Elongation at break = 1000% Tensile strength = 240 kPa Tensile modulus = 23 kPa of Compressive strength = 4 MPa	[106]
	IPNs	Physical cross‐linked hydrogel	PEDOT:PSS, PANI, Phytic acid	Compressive strength = 41.6 KPa	[107]
		Double‐network hydrogel	GelMA, ODEX rGO	Compressive strength = 200 kPa	[103]
*Electrical conductivity*	Electronic conductivity: Polymer‐based CHs	Physical cross‐linked hydrogel	PEDOT:PSS PANI Phytic acid	Energy density = 0.25 mWh cm^−3^	[107]
				Power density = 107.14 mW cm^−3^	
				Areal capacitance = 242.2 mF cm^−2^	
				Volumetric capacitance = 3.5 Fcm^−3^	
				Capacitance retention ratio = 80.8%	
	Electronic conductivity: Carbon‐based CHs	Double‐network hydrogel	GelMA, ODEX rGO	Ionic conductivity = 2.36 × 10^–2^ S m^–1^	[103]
	Electronic conductivity: MXene CHs	Nanocomposite hydrogel	PVA MXene	Performance as electrode = 0.5–2.0 Hz	[64]
				Durability with cycling = 3000 at 50% strain	
				Self‐healing efficiency = 15 s as electrode	
	Ionic conductivity: Electrolyte‐based hydrogel	Ionic hydrogel	OSA CMC AGO LiCl	Ionic conductivity = 1.15 S m^–1^	[104]
*Other properties*					
*Adhesion*	Redox activity of catechol/quinone groups	Catechol functionalized alginate (C‐Alg) CHs	EDC:NHS:Alginate DA Graphene	Adhesion energy: 1.79 J m^–2^	[12b]
		CHs	PEDOT LS‐PAm	Adhesive strength: 20–23 kPa	[108]
*Self‐healing*	Formation of imine bonds (Schiff base)	Polysaccharide‐based conductive ionic hydrogel	OSA CMC AGO LiCl	Self‐healing efficiency: 90% after 24 h	[104]
*Freezing and drying tolerance*	Hydrogen bonds between glycerin and water molecules	Conductive ionic PGT Organohydrogel	PAAc:gelatin TA in a water/glycerin AlCl^3+^	Original mechanical and conductive properties after 7 days at –14 °C	[67]
	Hydrogen bonds between glycerol and water molecules	Double‐cross‐linked CHs	PAAc:CMCs Ca^2+^ Water and glycerol	Reduction in conductivity (50%) and mechanical properties (20%) after 8 h at −20 °C	[109]
*Antibacterial activity*	Addition of polymer NPs as cross‐linking agents	NP‐cross‐linked hydrogel	PANI‐g‐MeGC Nps PAm	Against *Staphylococcus aureus*	[105]
	Addition of NPs	Non‐covalent cross‐linked hydrogel	PDA@Ag NPs PANI PVA	Against *Staphylococcus aureus* and *Escherichia coli*	[110]
*Biocompatibility*	Addition of polymer NPs as cross‐linking agents	NP‐cross‐linked hydrogel	PANI‐g‐MeGC Nps PAm	Viability of over 80% with NIH 3T3 cells	[105]
	Multiple non‐covalent cross‐linking	Non‐covalent cross‐linked hydrogel	PAm, CECT	Adhesion strength on pig skin = 113.2 kPa	[111]
	High‐density micelle cross‐linking	Non‐covalent cross‐linked hydrogel	PEG UPy	Adhesion strength on pig skin = 8 kPa	[106]
				Mouse fibroblasts cell (L929) viability over 95%	

AGO = Agarose, C2C12 = myoblast cell line, CMC = Carboxymethyl chitosan, CECT = Carboxyethyl chitin, DACS = Dialdehyde cholesterol‐modified starch, GelMA = Gelatin methacrylate, GF = Gauge Factor, IPNs = Interpenetrating polymer networks LS = Sulfonated lignin, L929 = Mouse fibroblasts cells, MeGC = Methacrylated glycol chitosan, MXene = Transition‐metal carbide/nitride, Nps = nanoparticles ODEX = oxidized dextran, OSA = Oxidized sodium alginate, PANI = Polyaniline, PAm = Polyacrylamide, PVA = Poly(vinyl alcohol), PANa = Sodium phytate, PDA@Ag NPs = polydopamine decorated silver nanoparticles, PEDOT:PSS = Poly(3,4‐ethylenedioxythiophene)‐poly(styrenesulfonate), PEG = poly(ethylene glycol), PPY = Polypyrrole, rGO = reduced graphene oxide, SA = Sodium alginate, UCMSCs = Umbilical cord mesenchymal stem cells, C2C12 = myoblast cell line, UPy = Ureido pyrimidinone.

### 4.1 Improvement in Mechanical Properties

Hydrogels with improved mechanical properties can be prepared by incorporating nanofillers^[^ ^100^, ^101^, ^102^, ^103^, ^104^, ^105^ ^]^ or using NPs and micelles as cross‐linkers.^[^ ^16^, ^106^, ^107^, ^108^, ^109^ ^]^ Another strategy to prepare hydrogels with improved mechanical properties involves the formation of interpenetrating networks.^[^ ^110^, ^111^, ^112^, ^113^, ^114^ ^]^

#### 4.1.1 Incorporation of Nanofillers

As discussed in Section 2.2.1, a common approach to prepare CHs is to incorporate nanofillers with conductive properties. These nanofillers are typically hydrophobic, whereas hydrogels are hydrophilic. The nanofillers can aggregate, which can deteriorate the mechanical properties of the hydrogels.^[^ ^112^ ^]^ The chemical modification of conductive nanofillers is a common approach to avoid aggregation and improve their affinity to hydrogels. Hydrophilic groups (–OH, –COOH, –F, and –O–) can be introduced onto nanofiller surfaces or edges using the oxidation or etching processes.^[^ ^112b^ ^]^ Another common strategy for nanofillers is loading them onto hydrophilic nanomaterials.^[^ ^112a^ ^]^

Different types of nanomaterials have been used as nanofillers, including carbon, metals, 2D nanomaterials, and metal oxide NPs. For example, gelatin‐functionalized oxidized carbon nanotubes (oxCNTs) were incorporated into PAm hydrogels. In this study, cross‐linked PAm comprised the backbone of the final hydrogel, and oxCNTs were used as nanofillers to improve the mechanical properties. The resulting PAm/oxCNT hydrogel showed high stretchability (>700%), high tensile strength (0.71MPa), and super durability (over 300 cycles).^[^ ^113^ ^]^

In some cases, more than one nanofiller can be incorporated into a hydrogel. For example, cellulose nanofibers (CNFs) were introduced into PAm hydrogels to improve their mechanical properties. Simultaneously, CNFs have been used as a dispersion agent for CNTs, which are electrically conductive nanofillers. With the incorporation of 1 wt.% CNFs and 1 wt.% CNTs into the PAm hydrogel, the resultant PAm/CNF/CNT hydrogel exhibited remarkably improved mechanical properties. The fracture tensile strength and elongation at break increased from ≈0.1 to ≈0.32 MPa and from ≈58% to 140%, respectively.^[^ ^114^ ^]^

The in situ synthesis of conductive nanofillers in hydrogels has also been reported.^[^ ^115^ ^]^ Cellulose nanocrystals (CNCs) previously coated with tannic acid (TA) were used to immobilize and stabilize Ag–NPs. TA is a water‐soluble natural polyphenol capable of reducing silver ions (Ag^+^) to Ag–NPs owing to its reductive phenolic hydroxyl groups. This avoids the use of environmentally harmful agents or hydrothermal treatments, which are usually required to join metal NPs in cellulose. Additionally, the catechol groups of TA facilitate the dispersion of Ag onto the CNCs and allow reversible cross‐linking with the polymer matrix. The resulting Ag–NPs stabilized with TA‐coated CNCs were incorporated into the PVA hydrogel. This hydrogel exhibited enhanced conductivity (4.61 S m^−1^) and stretchability (>4000%) and remarkable improvements in mechanical properties, particularly the tensile strength (from ≈70 to 246 kPa) and fracture strain (from ≈1700% to 4100%). In addition, it exhibited excellent antibacterial properties and repeatable self‐healing ability.^[^ ^116^ ^]^

#### 4.1.2 Cross‐Linkers Used for the Preparation of Hydrogels

**Figure 9:**
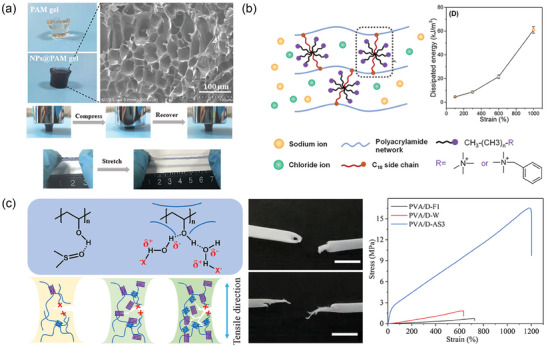
a) Photographs of PAm and PANI NPs cross‐linkers in PANI NPs‐PAm hydrogels along with an SEM image. Reproduced with permission.^[^ ^105^ ^]^ Copyright 2022, Wiley‐VCH. b) Illustration of P(Am‐Hb/CM) and the dissipated energy at various strains. Reproduced with permission.^[^ ^119^ ^]^ Copyright 2022, Elsevier. c) Schematic illustration of tensile fracture in PVA samples, and the tensile stress‐strain curves. Reproduced with permission.^[^ ^120^ ^]^ Copyright 2022, Elsevier.

Some NPs can serve as cross‐linkers in hydrogel preparation. For example, PANI NPs were synthesized by grafting PANI onto methacrylated glycol chitosan (MeGC). These Me‐PANI NPs were introduced during the polymerization of PAm and served as cross‐linking agents in the PAm hydrogels^[^ ^105^ ^]^ (**Figure** **9**a). PAm hydrogels prepared using 5 mg mL^−1^ of PANI NPs (PANI–PAm) showed higher resistance to stretching than PAm hydrogels. The maximal elongation of the PANI–PAm hydrogels was 400% at 50 kPa and that of the PAm hydrogels was 180% at 18 kPa.

Dialdehyde cholesterol‐modified starch nanoparticles (DACSNPs) were used as cross‐linkers to prepare collagen hydrogels. These NPs were synthesized by the self‐assembly of amphiphilic dialdehyde cholesterol‐modified starches. The incorporation of DACSNPs as cross‐linking agents in collagen hydrogels remarkably improved the mechanical strength and morphology, increasing the maximum compressive strength to 70.8 kPa (compared with 7.8 kPa for the collagen hydrogel) and leading to the formation a uniform 3D porous structure. Additionally, the Young's modulus of the DACSNP‐Col hydrogel was 6.5 kPa, while that of the collagen hydrogel was 2.1 kPa. The resulting hydrogel was proposed as a potential scaffold for tissue engineering.^[^ ^117^ ^]^

An improvement in the resistance to stretching and deformation can lead to a decrease in the toughness of hydrogels.^[^ ^106^ ^]^ The use of micelles as cross‐linkers appears to be a suitable option to avoid a decrease in toughness because micelles can easily deform under a load and are capable of distributing stress. Micelles are soft particles in the range of 10–100 nm that self‐assemble from amphiphilic polymers in an aqueous environment.^[^ ^118^ ^]^ Yang et al. prepared hydrogels using C8‐ONH_2_ micelles.^[^ ^106^ ^]^ The hydrogel formed by C8‐ONH_2_ micelles was linked to the PEG chains through oxime bonds. The resultant hydrogel containing 90 wt% water showed non‐swelling behavior, quick self‐recovery, and excellent mechanical properties, such as 4 MPa compressive strength, 240 kPa tensile strength, and 1000% stretchability. In another study, Zeng et al. fabricated a conductive poly(acrylamide) hydrophobic monomer/cationic micelle (P(Am‐Hb/CM)) hydrogel with self‐healing, antimicrobial, and mechanical properties using cationic micelles cross‐linked in a PAm network^[^ ^119^ ^]^ (Figure 9b). For this purpose, the hydrophilic monomer Am was copolymerized with the hydrophobic monomer stearyl methacrylate (also known as C18), which was preserved in cationic micelles comprising benzalkonium chloride and cetyltrimethylammonium chloride. The resulting hydrogel exhibited a high stretchability of >1100%, elasticity of >83%, recovery from 1000% tensile strain, and adequate electrical properties owing to the multiple dynamic and reversible non‐covalent interactions.^[^ ^119^ ^]^

#### 4.1.3 IPN

The preparation of IPN hydrogels allows for the combination of good electrical conductivity and the mechanical properties of two different polymer networks. The preparation of cross‐linked double networks is a common strategy used to address the poor mechanical properties of certain conductive polymer networks. The idea is to combine the good conductivity of a network, which is usually soft and weakly cross‐linked, with the rigidity of another network, which is well cross‐linked, to improve the mechanical properties of the IPN hydrogel. For instance, a double‐network PEDOT/PANI hydrogel was produced by integrating two conductive polymers, PANI and PEDOT, using phytic acid as a cross‐linker. It showed improved mechanical properties with a compressive strength of 41.6 kPa, which is higher than that of PEDOT hydrogels (4.5 kPa). In addition, it exhibits excellent conductive properties, making it a promising material for use in flexible solid‐state supercapacitors.^[^ ^107^ ^]^

The inhomogeneity between these networks may cause an imbalance in the energy‐dissipation distribution. Therefore, an increase in stiffness could deteriorate the toughness of the material, and vice versa.^[^ ^121^ ^]^ To address this, the use of homogeneous networks has been proposed for fabricating stiff, tough hydrogels with uniform structures and adequate energy‐dissipation mechanisms.^[^ ^120^ ^]^ Amphiphilic PVA can form non‐covalent bonds, such as hydrogen bonds and hydrophobic interactions, with other PVA chains. A rough CH was created using homogeneous PVA networks and a one‐step solvent‐exchange strategy.^[^ ^120^, ^122^ ^]^ PVA chains are self‐assembled and cross‐linked by PVA crystalline domains and hydrophobic interactions. As shown in Figure 9c, the resulting hydrogel exhibited remarkable mechanical properties with a tensile strength of 16.54 MPa, elongation at break of 1200%, Young's modulus of 1.56 MPa, and toughness of 111.21 MJ m^−3^.^[^ ^120^ ^]^ The hydrogel was used in the construction of a novel supercapacitor, exhibiting a superior capacitance (156.50 mF cm^−2^ at 1.0 mA cm^−2^).

Sun et al. prepared hydrogels constructed from two homogeneous networks. Specifically, they used two cross‐linkers for the synthesis of a PAm nanocomposite hydrogel: the conventional organic cross‐linker MBAA, and a second cross‐linker, calcium hydroxide nanospherulites (CNS).^[^ ^123^ ^]^ The CNS acted as a cross‐linker of the bare polymer chain and connected adjacent MBAA clusters. The resulting hydrogel exhibited remarkable mechanical properties, including high compressive stress (220 MPa) and high stretchability (2200%) under high tensile stress (920 kPa). This hydrogel also exhibited self‐recoverability and maintained similar mechanical properties after many cycles of compression.^[^ ^123^ ^]^

### 4.2 Improvement of Functionalities

#### 4.2.1 Adhesion

Adhesive proteins are used to impart adhesion to the hydrogels. Adhesive proteins in mussel byssus have inspired the development of a variety of adhesive substances. The capacity for adhesion of the mussel byssus is due to the proteinaceous excretion of 3,4‐dihydroxyphenyl‐L‐analine (DOPA). Catechol groups derived from DOPA were used to adhere to alginate hydrogels. Naguib et al. reported the functionalization of alginate with catechol groups. The resulting CHs exhibited an adhesion energy of 1.79 J m^−2^.^[^ ^12b^ ^]^ Conductive hydrophilic NPs were synthesized using PEDOT and sulfonated lignin (LS). Lignin is hydrophilic and adhesive owing to the redox activity of its catechol/quinone groups. The PEDOT/LS NPs were used as nanofillers in the PAm network. The resulting hydrogel (PEDOT/LS‐PAm) with 3 wt.% NP content showed a long‐term and repeatable adhesiveness, with adhesive strengths in the range of 20–23 kPa for different materials such as steel, glass, and porcine skin.^[^ ^108^ ^]^ TA has also been used to adhere to hydrogels owing to the presence of catechol groups in its molecular structure. The incorporation of TA‐CNTs into the PAm/SA hydrogel resulted in a high and repeatable adherence. The maximum adhesion strengths of PAm/SA/TA‐CNT hydrogels to wood, glass, aluminum, polytetrafluoroethylene (PTFE), and porcine skin were reported to be 43, 17, 15, 5, and 1.2 kPa, respectively.^[^ ^124^ ^]^

#### 4.2.2 Self‐Healing Properties

Self‐healing refers to the ability of a material to repair itself after external damage while preserving its original functionalities. Self‐healing allows for lifespan prolongation, reusability, and enhanced responsiveness to environmental stimuli.^[^ ^125^ ^]^ Self‐healing properties can be obtained by incorporating reversible bonds or supramolecular interactions among the polymer functional groups.^[^ ^112a^ ^]^ These bonds break when the hydrogel fails. However, these bonds were reconstructed when the damaged surfaces were in contact. This property can be affected by ambient conditions such as temperature and pH.^[^ ^104^ ^]^ Self‐healing hydrogels have potential applications in many fields, such as repairable circuits,^[^ ^126^ ^]^ wearable electronic devices,^[^ ^1a^ ^]^ electronic skin,^[^ ^127^ ^]^ multifunctional sensors,^[^ ^128^ ^]^ tissue engineering,^[^ ^129^ ^]^ and biomedicine.^[^ ^24^ ^]^

An imine bond, also known as a Schiff base, is a functional group formed by the reaction between the amine and aldehyde groups. The reversible nature of imine bonds allows the fabrication of self‐healing systems. SA and CMC have been used to prepare self‐healing hydrogels. Aldehyde groups were incorporated through the oxidation of SA. OSA provided these networks with the capability to form a Schiff base structure between OSA and CMC. The resulting hydrogel displayed complete self‐healing capacity after 24 h. The self‐healing efficiency of the hydrogel was approximately 90% according to the evaluation of the compressive stress–strain curves before and after self‐healing.^[^ ^104^ ^]^

#### 4.2.3 Freezing and Drying Tolerance

The capacity of hydrogels to tolerate high and low extreme temperatures while maintaining their integrity and properties has expanded their application to harsh environments. Several strategies have been investigated to maintain the structural properties of hydrogels at extreme temperatures, including the use of polymers with catechol groups, and water and glycerol as binary solvents. For example, an anti‐freezing and anti‐drying hydrogel was prepared using PAAc, gelatin, and AlCl^3+^ in a dispersion medium composed of water and glycerin. After long‐term exposure to a subzero temperature (–14 °C), this hydrogel maintained its conductivity, adhesion strength, and strain sensitivity. It also exhibited high transparency (85% transmittance) and high stretchability (1200%).^[^ ^67^ ^]^ A double‐network hydrogel was prepared using PAAc and carboxymethylcellulose. Water and glycerol binary solvents were introduced into the hydrogels to impart anti‐freezing properties. The resulting hydrogel maintained its integrity after exposure to –20 °C for 8 h.^[^ ^109^ ^]^

#### 4.2.4 Antibacterial Properties

The incorporation of metal NPs can endow CHs with antibacterial properties. Lin et al. incorporated silver NPs into polydopamine (PDA)/PANI/PVA hydrogels. The final products showed broad antibacterial activity against both gram‐negative and gram‐positive bacteria.^[^ ^110^ ^]^ The cationic polyelectrolyte poly(diallyl dimethyl ammonium chloride) was grafted from bacterial cellulose (BC) nanofibers via SI‐ATRP (BCD) into a PDA/PAm hydrogel. The resulting BCD/PDA/PAm hydrogels exhibited long‐lasting antibacterial properties and rapid wound healing.^[^ ^130^ ^]^

Antibacterial activity can also be imparted to hydrogels by the addition of quaternary ammonium salts. A hydrogel with antibacterial activity was prepared by mixing a quaternized chitosan solution with benzaldehyde‐terminated poly(ethylene oxide)‐b‐poly(propylene oxide)‐b‐poly(ethylene oxide). The hydrogel exhibited excellent antibacterial properties.^[^ ^131^ ^]^

Hydrogels used in photothermal therapy (PTT) have exhibited promising applications as antibacterial wound dressings. PTT is based on the use of rapid hyperthermia generated by photothermal agents when exposed to near‐infrared radiation, which kills bacteria.^[^ ^9^ ^]^ These agents are used as fillers or cross‐linking agents in hydrogels to impart photothermal antibacterial properties. The most common antibacterial agents are gold‐, carbon‐, and graphene‐based nanomaterials^[^ ^132^ ^]^ as well as PDA and PANI.^[^ ^29^, ^133^ ^]^ Among these, PANI is widely used in PTT owing to its high photothermal conversion efficiency and biocompatibility. For example, PANI was grafted onto MeGC to develop an amphiphilic copolymer, which was then used to prepare NPs via sonication in an aqueous solution. These NPs endowed the PAm hydrogels with antibacterial properties. The PAm hydrogels containing these NPs showed a reduction in bacterial‐colony‐forming units (CFU), that is, from 6 to 1 CFU mL^−1^, compared with blank PAm hydrogels.^[^ ^105^ ^]^

#### 4.2.5 Biocompatibility

Natural polymers can also be used to prepare CHs. For example, chitin, a natural polymer extracted from shrimp and crab exoskeletons, has been used to prepare CHs. Natural polymer hydrogels were prepared by polymerizing Am in the presence of carboxyethyl chitin (CECT). This hydrogel displayed high stretchability (1586%).^[^ ^111^ ^]^ Other natural polymers used to prepare CHs include SA, gelatin, CS, cellulose, hyaluronic acid, and agar; however, these materials exhibit poor mechanical properties. A common approach for addressing this problem is the use of a second polymeric network to synthesize double‐network hydrogels.^[^ ^134^ ^]^ Agarose has also been used to improve the mechanical properties of natural polymer hydrogels.^[^ ^135^ ^]^ For instance, CHs with inherent biocompatibility and good self‐healing, mechanical, and conductive properties have been successfully prepared by combining and dissolving natural polysaccharide polymers (OSA, CMC, and AGO) in an LiCl solution. The resulting hydrogels were used to fabricate wearable devices.^[^ ^104^ ^]^

## 5 Application of CHs for Wearable Devices

### 5.1 CH‐Based Strain Sensors

The most promising materials for strain‐sensor applications are based on CHs because such materials can effectively change their electrical properties and polymer network structure in response to external stimuli. Such changes selectively cause the conductive network to become denser or sparser in response to applied stimuli such as pressure and strain, resulting in changes in electrical resistance or conductance. The sensing performance of strain sensors is also dependent on the synergistic effect between the hydrogel matrix and the conductive network. The network structure of hydrogels greatly affects critical parameters such as the sensitivity and performance of CH strain‐sensor devices.^[^ ^136^ ^]^ The two main factors used to measure the sensitivity are the gauge factor and limit of the changed conductivity.

**Figure 10:**
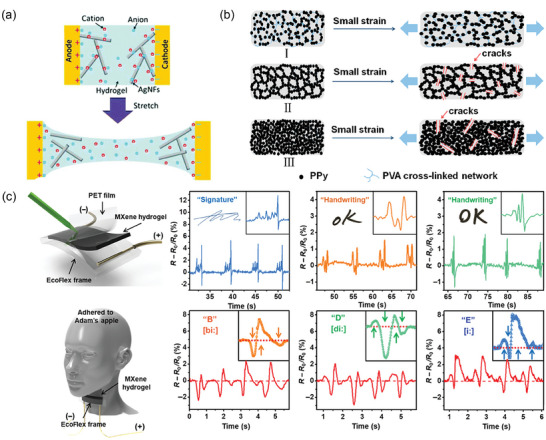
a) Schematic of strain sensor based on brick mortar structure and CHs. Reproduced with permission.^[^ ^8b^ ^]^ Copyright 2019, Royal Society of Chemistry. b) Piezoresistivity mechanism of a PVA‐PPy strain sensor in response to small strain. Reproduced with permission.^[^ ^138^ ^]^ Copyright 2020, American Chemical Society. c) Strain sensors based on PVA/MXenes material and examples of signature sensing, handwriting, voice sensing, and changes in resistance in response to similar words. Reproduced with permission.^[^ ^139^ ^]^ Copyright 2018, American Association for the Advancement of Science.

Chu et al. prepared a strain sensor from a composite composed of silver nanofibers (AgNFs) embedded in chemically cross‐linked alginate and covalently cross‐linked PAm.^[^ ^8b^ ^]^ As illustrated in **Figure** **10**a, the strain–capacitance response of the sensor was achieved through a geometrical effect. In the initial condition, the electric double‐layer capacitance was high owing to the presence of the embedded AgNFs. By contrast, the AgNF contact with the electrodes was removed when the sensor was stretched, decreasing the capacitance.

The working range and sensitivity of CH‐based strain sensors are often improved by integrating several sensing mechanisms, among which the piezoresistive mechanism is the most important.^[^ ^137^ ^]^ The mechanism of the piezoresistive PVA‐PPy‐CH strain sensor is illustrated in Figure 10b.^[^ ^138^ ^]^ When a small strain was applied, the PVA network deformed, leading to the development of local cracks in the path of the CPs and increased resistance. In the piezoelectric mechanism, the conductor content plays an important role in the sensing performance. An excessive or low number of conductors in the hydrogel matrix of the CPs easily destroy or blocks the conductive paths. This reduces the resistance and sensitivity of the material, even under a small strain. A sufficient conductor content in the CH network allows for high sensitivity, good repeatability, and reversible stretching and releasing processes.

MXenes have been combined with different types of polymers to develop wearable strain sensors with excellent sensitivities. Tang et al. prepared a multifunctional epidermal sensor based on MXene (Ti_3_‐C_2_‐T_x_) and PAAc that exhibited superior properties in terms of stretchability, self‐healing degradability, sensitivity, and a fast response to human motion.^[^ ^140^ ^]^ A CH‐based strain sensor consisting of MXenes and a double‐network hydrogel of PAm and SA exhibited good tensile strength and excellent electrical stability.^[^ ^141^ ^]^ Developed by Alshareef et al., PVA hydrogels containing MXenes (in the form of nanosheets) were highly stretchable and exhibited instantaneous self‐healing properties and better adhesion to human skin.^[^ ^139^ ^]^ The addition of nanosheets considerably increased the strain capacity of the hydrogel, and without any complex circuit design, it was used as a sensing film for advanced sensing applications, such as signature recognition, handwriting, and vocal recognition (Figure 10c). MXene nanosheets have been identified as promising materials for the development of wearable electronics, point‐of‐care testing, and soft robotics for human healthcare monitoring.

### 5.2 Electronic‐Skin Devices

Electronic skin and e‐skin are self‐healing electronics that mimic the functionalities of human skin. These materials are flexible and stretchable and can perceive subtle stimulus signals.^[^ ^142^ ^]^ Highly flexible and biocompatible e‐skins have recently garnered significant research interest in the development of prosthetics, human–machine interfaces, human health detection, and soft robotics. To integrate e‐skin devices with human skin for practical applications, the features of multimodal sensing and flexibility must be effectively integrated. The conversion of external stimuli to electrical signals is one particular feature that is vital for the identification of the location of the device.^[^ ^143^ ^]^ CHs are highly transparent with outstanding conductive properties under minor stress–strain loops and are potential candidates for the design and development of e‐skin devices.^[^ ^7^, ^144^ ^]^ Furthermore, these materials strongly adhere to human tissues, which reduces the resistance of the interfaces and the artifacts developed during motion. Devices for attachment to human skin are fabricated from a thin CH film with two or three electrodes. Given their properties, CHs have been widely used in the development of e‐skin microsensors with versatile properties such as good adhesion, high elasticity, quick response, good compatibility, and human‐skin‐like performance.

Fan et al. developed a CH‐based skin‐biocompatible e‐skin device that combined PVA, phytic acid, polypropylene glycol, and gelatin.^[^ ^145^ ^]^ This combination of materials provided enriched hydrogen bonds, resulting in superior properties such as good adhesion, high transparency, facile detachability, antimicrobial resistance, and recyclability. This material could also recognize electrophysiological signals during human motion. These properties allow for easy adaptability of the material/device during stretching and bending of the wrist and are better than those of commercially available hydrogels.

### 5.3 CH‐Based TENGs

In 2012, Lin Wang et al. invented the triboelectric nanogenerator (TENG) as a result of coupling between electrostatic induction and frictional electric effects.^[^ ^146^ ^]^ TENGs are based on the triboelectric effect, which is related to the generation of electricity owing to contact between two different materials. When the tribo‐positive layers come into contact with the tribo‐negative layer, contact electrification occurs between the two layers, generating an equal number of opposite charges on the surface of the layers. When the two tribolayers are separated, an electrostatic potential difference is created on their surfaces. These nanogenerators can harvest mechanical energy from waves, wind, human body movements, and respiration. The attractive features of these devices make them promising for applications such as artificial intelligence,^[^ ^147^ ^]^ wearable electronic devices,^[^ ^148^ ^]^ biomedical devices,^[^ ^149^ ^]^ and the Internet of Things.^[^ ^150^ ^]^ Rigid materials are widely used as electrode materials in conventional TENGs; however, they perform poorly under large strains owing to the rigidity constraints of the electrode material.^[^ ^151^ ^]^ To overcome this issue, tunable and electrically CHs with or without conductive fillers, such as metallic NPs, salts, carbon materials, and conductive polymers, have been used as flexible and stimuli‐responsive electrode materials.^[^ ^152^ ^]^

In hydrogel‐based TENGs, the CHs are typically encapsulated by elastomers and connected as electrodes using wires.^[^ ^153^ ^]^ Hydrogel electrodes can be ionic or electronic with different charge‐transfer properties. For ionic hydrogel electrodes, the negative ions cause redistribution of the ions in the hydrogel, and the positive ions move toward the negative charges and vice versa, causing polarization and output performance. When the two tribolayers come into contact, the flow of positive and negative ions is reversed by the simultaneous generation of output power. The working principle of hydrogel TENGs relies on the transfer of electrons to generate output power, and the process is similar to that for ionic hydrogel electrodes. The important hydrogel electrode materials used in the fabrication of the hydrogel‐based TENGs are listed in **Table** **2**.

**Table 2:** Hydrogel electrode materials and output performance of TENGs.

Hydrogel	Network structure	Open‐circuit voltage [V]	Short‐circuit current	Peak power density	References
PVA	SN	200	22.5 µA	‐	[154]
PAm	SN	312	32.4 µA	2.7 W m^−2^	[155]
Cellulose	SN	187	0.51 µA	‐	[42]
PAAc	SN	180	65 µA	625 µW m^−2^	[156]
HA	SN	20	0.4 µA	5.6 mW m^−2^	[8a]
PAm/HEC	MN	285	15.5 µA	626 mW m^−2^	[157]
PAm/PDA	MN	230	12 µA	‐	[158]
PVA/SA	MN	204	18 µA	0.98 mW m^−2^	[152b]
PAm/Alginate	MN	70	0.5 µA	135 mW m^−2^	[159]
PAm/Cyclodextrin	MN	95	10 µA	0.64 mW m^−2^	[160]
PAm/clay	NC	89	16 µA	710 mW m^−2^	[3]
PVA/MXene	NC	230	1.2 µA	0.33 W m^−2^	[161]
PAm/Graphene	NC	40	1.6 mA	0.3 W m^−2^	[162]
Cellulose/ZnO	NC	58	5.78 µA	42 mW m^−2^	[163]

SN = single network, MN = multi‐network, NC = nanocomposite.

#### 5.3.1 Self‐Powered Sensors

**Figure 11:**
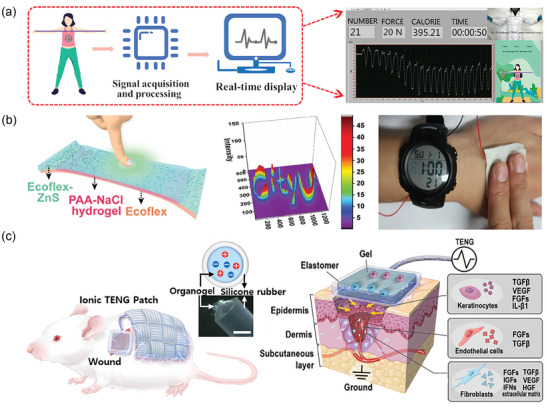
a) Schematic of a self‐powered, smart elastic‐band system including the signal acquisition and processing system, and a real‐time output interface displaying the potential of this TENG for human‐motion monitoring. Reproduced with permission.^[^ ^164^ ^]^ Copyright 2021, American Chemical Society. b) Demonstration of the smart‐skin device with the image and digital resolution of light‐emitting letters “CityU” and the electronic circuit of the self‐charging system used to power an electronic watch. Reproduced with permission.^[^ ^156^ ^]^ Copyright 2019, Wiley‐VCH. c) Schematic representation of accelerated wound healing owing to secretion of biomolecules and formation of new tissues under a self‐powered device using ionic TENG. Reproduced with permission.^[^ ^166^ ^]^ Copyright 2021, Elsevier.

Flexible sensors can be fabricated from materials that can extend without loss of properties, and TENGs based on hydrogel electrodes provide good flexibility and electrical conductivity. In the fields of motion monitoring, haptic perception, and human–computer interactions, a self‐powered TENG based on a multinetwork structure developed by Wang et al. exhibited good linearity and reliability under external tensile stress and recognized the motions of various parts of the human body.^[^ ^164^ ^]^ The potential application of this device in human‐motion monitoring and energy‐harvesting applications was demonstrated through signal acquisition and a real‐time software output interface using a self‐powered smart elastic band (**Figure** **11**a).

A dual‐electrode TENG based on PDMS and an ionic hydrogel prepared by Wang et al. could recognize the pressure change as a function of different stretching profiles.^[^ ^165^ ^]^ The device exhibited good reliability in measuring pulse beating, breathing, and human motion, making it a potential candidate for human healthcare applications. Developed by Wang et al., a self‐powered sensor containing an encapsulated nanocomposite hydrogel in an elastomer generated different output voltages as functions of strain and amplitude changes.^[^ ^161^ ^]^ This self‐powered tactile sensor recognizes letters and words written on the TENG, showing great potential for applications in stroke‐recognition high‐precision wearable devices.

#### 5.3.2 Biomechanical Energy‐Harvesting Materials

Hydrogel‐based TENGs are widely used in the fabrication of wearable electronic devices to harvest biomechanical energy because electrical energy can be harvested when these materials are mechanically deformed. By adding Cu‐doped zinc sulfide particles to a biodegradable and compostable Ecoflex, a TENG with mechanoluminescent properties was developed.^[^ ^156^ ^]^ (Figure 11b). This device has multidimensional properties such as long stability, extended pressure detection (limit of 0.58 kPa), and light‐emitting response. This study lays a foundation for the development of multifunctional self‐powered epidermal electronics. Devices based on poly(*N*‐acryloyl glycinamide) and PAm developed by Wang et al. exhibited sol–gel transitions owing to changes in the hydrogen‐bonding capacity as a function of the external temperature.^[^ ^167^ ^]^ These materials are extruded into fibers, coated with poly(methyl methacrylate), and woven into fibers to power electronic watches. Inspired by the micropatterns of octopus tentacles, Li et al. developed a device that could harvest energy from water.^[^ ^153^ ^]^ Ecoflex was used as the triboelectric layer, on which micropatterns of a polyampholyte hydrogel were created to enhance the output of the TENG. This device collects energy from waves as water falls against a smooth glass substrate.

### 5.4 Wearable Devices for Drug Delivery

Regulated and on‐demand delivery of therapeutics to the target site is a major hurdle in drug‐delivery systems. CHs have gained significant interest owing to their low electrical potential, which can be exploited for controlled on‐site release of therapeutics. PPy‐based TENGs have been used to efficiently deliver neurotrophin‐3, nerve growth factors, and brain‐derived neurotrophic factors for neuronal development and differentiation.^[^ ^168^ ^]^

The use of hydrogel‐based TENGs in biomedical applications has gained considerable attention owing to their biocompatibility and ease of fabrication into wearable devices. These devices stimulate and accelerate wound healing via electrostatic simulations. Sun et al. developed a stretchable organohydrogel in silicone rubber.^[^ ^166^ ^]^ This wearable generator device on a hydrogel electrode was prepared as a patch for wound dressing (Figure 11c). Physical movement generates a voltage, which in turn generates an endogenous electric field in the wound patch. This stimulated the wound, promoting healing at a significantly higher rate than the wound patch made from an ordinary hydrogel that was not electrically conductive. A soft hydrogel patch with side‐by‐side electrodes was developed by Wang et al. for noninvasive iontophoresis.^[^ ^5b^ ^]^ A proof of concept was demonstrated using organic dyes as model drugs and pig skin as a wearable device. This study extends the scope of TENGs as cost‐effective materials for noninvasive and electrically stimulated iontophoretic drug delivery of therapeutic agents.

## 6 Seamless Integration of Conductive Hydrogels for Wearable Applications

Most wearable systems include sensors and functional devices that are designed to be integrated into a part of the body. For instance, direct integration with the epidermis can provide a seamless integration. This strategy requires integrating devices with the soft and curvilinear surfaces of the human body.^[^ ^169^ ^]^ Stretchable electronic materials are required to achieve this type of integration. Hydrogels are materials that can provide seamless, noninvasive interfaces that remain robust during natural movements and associated biological processes. In addition, they closely mimic the mechanical, chemical, and optical properties of biological tissues.^[^ ^170^ ^]^ These bioelectronic wearable devices should have certain desirable characteristics, such as a skin‐like Young's modulus (low modulus < 225 KPa), high stretchability (strain > 100%), and high conductivity under heavy load. For example, Wang et al. developed an elastomer integrated with a conductive hydrogel that seamlessly integrates with the skin, featuring shape‐adaptive characteristics and low contact impedance.^[^ ^171^ ^]^ This conductive hydrogel enables real‐time, high‐quality detection of electrocardiogram signals by functioning as a flexible electrode.

Lv et al. synthetized a conductive P(BHMP‐AM)‐Zn^2+^ hydrogel material in situ via a one‐pot method using acrylic monomer derivatives, acrylamide, and zinc ions.^[^ ^172^ ^]^ This material had skin‐like properties to use it as a bio‐sensor to study joint and another body‐parts activity, it also showed a tensile strain > 2000% a compressive rate of 17 MPa at a compressive stress of 85%.

The use of electronic textiles is another strategy to seamlessly integrate wearable devices. In recent years, there has been a noticeable rise in the utilization of electronic textiles, marking a shift from heavy metallic materials to lightweight textile alternatives.^[^ ^173^ ^]^ The emergence of electronic textiles, or e‐textiles, involves integrating electronic components into traditional fabrics. This integration enables the fabrics to perform various functions in conjunction with contemporary technologies, such as displays, sensors, and controllers.^[^ ^174^ ^]^ Four different levels of integration have been defined for e‐textiles: removable, attached, mixed solution, and full textile solution.^[^ ^175^ ^]^ At the initial level, the electronic device is detachable and added to the textile as a separate unit. The second level involves integrating an electronic device in a way that prevents its removal without causing damage to the textile. The third level consists of a combination of attachable and non‐attachable devices. Lastly, the fourth level signifies a comprehensive integration where all components are made to resemble textile materials8.

In this context, conductive hydrogels can be used to fabricate seamless integrated e‐textiles. For example, Wang et al. used conductive hydrogels as fibers for the manufacturing of smart textiles, capitalizing on its highly stretchable property.^[^ ^167^ ^]^ The resulting hydrogel fiber exhibited a tensile strength of 2.27 MPa, stretchability of 900%, high conductivity of 0.69 S m^−1^, and self‐healing capability. This conductive fiber was then used to weave a textile for creating a strain sensor and a TENG. Li et al. developed conductive ionic hydrogel fibers based on polyimide salt, these fibers showed a conductivity of 21 mS cm^−1^, a tensile strength of 2.5 MPa and breaking elongation of 215%.^[^ ^176^ ^]^ The fibers were used to weave a textile with good wearable properties and use it as a strain sensor for biomechanic measurements.

## 7 Conclusion

Based on recent progress, CHs are promising for smart wearable devices owing to their advantages such as soft modulus and biocompatibility, which allow for comfortable long‐term wear on the human body for monitoring health conditions and motions, delivering drugs, or harvesting energy. CH preparation involves various aspects, including dispersing conducting fillers in the hydrogel network, swelling in the electrolyte, and the formation of a CP network.

Most recent developments of CHs are in smart batteries, smart electrodes, for enhanced bioelectronics, medical imaging systems, and transdermal drug delivery systems. As the demand for wearable bioelectronics in the healthcare sector continues to grow, there are a number of challenges. One of the main challenges is the development of single‐networkCHs with combined properties such as biocompatibility, high mechanical strength, antimicrobial resistance, conductivity and sensitivity. Another challenge is the limitation in performance of the device which is influenced by the type of conductive component of the hydrogel such as ionic, conductive‐polymer, carbon‐based or metallic‐based. The power consumption of the integrated circuit, input energy sources, long‐term stability of the material, and response‐sensitivity also needs to be considered carefully in the design aspects of these devices. Recently, 3D printing technology has gained importance in the fabrication of wearable sensors. For this to be successful, uniformity of the hydrogel or nanocomposite during printing is a prerequisite. For example, the current 3D print techniques such as laser‐based printing, extrusion‐based printing and ink‐jet‐based printing requires different material requirements for practical applications.

These limitations could be overcome through process optimization in the development of new types of CHs and novel conductive fillers. Furthermore, in wearable devices the sensor comfort (interface between the conductive hydrogel and human skin) and wearability needs to be optimised. The manufacture of these new materials from natural polymers (biomass‐based) can improve the biocompatibility and biodegradability. This can be achieved through increase in cross‐link density to improve the mechanical properties, enhancing the frost resistance through metal ions, and enhancing the self‐healing properties through dynamic or non‐dynamic covalent bonds. However, achieving all of these multiple properties in a single system still remains a challenge, and overcoming these limitations will open up and widen the application market of conductive hydrogels in wearable bioelectronics, energy storage devices, and soft‐robot sensors.

By overcoming these challenges, CHs can revolutionize the field of wearable devices, enabling the development of comfortable, high‐performance, and biocompatible systems for various applications such as biosensors, energy harvesting, and electronic skin. Continued research and development efforts are necessary to optimize preparation processes, enhance mechanical properties, improve conductivity, and ensure biocompatibility, which can be achieved by designing a novel network structure, forming an efficient percolation network, swelling in highly conductive electrolytes, and incorporating natural polymers.

## Conflict of Interest

The authors declare no conflict of interest.

## Data Availability

The data described in the article are available at https://doi.org/10.5281/zenodo.10008178. We would appreciate if other researchers could benefit from our literature and results. This will foster discussions and collaboration among scientists worldwide.
